# Prediction of protein architectures involved in the signaling-pathway initiating sporulation in Firmicutes

**DOI:** 10.1186/s13104-019-4712-3

**Published:** 2019-10-23

**Authors:** Paola Martinez-Amador, Nori Castañeda, Antonio Loza, Lizeth Soto, Enrique Merino, Rosa Maria Gutierrez-Rios

**Affiliations:** Departamento de Microbiología Molecular, Instituto de Biotecnología, Universidad Nacional Autónoma de México, Apdo. Postal 510-3, 62250 Cuernavaca, Morelos Mexico

**Keywords:** Sporulation, Firmicutes, Protein architecture, Orthologs

## Abstract

**Objectives:**

Like many other proteins, those belonging to the signal transduction cascade initiating sporulation (Spo0 pathway) have conserved protein domains (Capra and Laub in Annu Rev Microbiol 66:325–47, 2012). Improvements in bioinformatics applications to discover proteins involved in the initiation of the sporulating cascade in newly sequenced genomes is an important task that requires rigorous comparative genomic methods and manual curation to identify endospore-forming bacteria. This note aims to present a collection of predicted proteins involved in the Spo0 pathway found in the proteomes of fully sequenced and manually curated endospore-forming Firmicutes species. This collection may serve as a guide to conduct future experiments in endospore formers in genomic and metagenomic projects.

**Data description:**

Similar to the report of Davidson et al. (PLoS Genet 14:1–33, 2018), we used Pfam profiles (El-Gebali et al. in Nucleic Acids Res 47:D427–32, 2019) defining each protein and the genomic context surrounding the query gene to predict probable orthologs of the Spo0 pathway in Firmicutes. We present in this note a collection of 325 Firmicutes species organized by phylogenetic class and classified as spore formers, non-spore formers or unknown spore phenotype based on published literature, for which we predicted probable orthologs defining the signal transduction pathway initiating sporulation.

## Objective

Comparative genomic analysis is a valuable tool to explore new genomes and metagenomes to search for functional processes, phylogenetic relationships, and evolutionary traits among organisms. An example of this comparative analysis is finding that proteins involved in the Spo0 pathway constitute of at least a sensor kinase, the phosphotransferases Spo0F and Spo0B, and the response regulator Spo0A [1], were suggested to be ancestral since it was found not only in Bacilli but also in some Clostridia [2]. While an original article from our group that describes the distribution and prevalence of the Spo0 pathway and also confirms that this pathway is ancestral was under review, the results mentioned above were published by Davidson and collaborators using a set 84 Firmicutes [2]. Therefore, in this note, we provide a more extensive list of manually curated Firmicutes possessing different forms of the Spo0 pathway initiating sporulation in Firmicutes.

## Data description

The data are a collection of predicted protein architectures defining the proteins shaping the Spo0 pathway in Firmicutes. As a guide, we used the proteins shaping the sporulation cascades in the model organisms shown in Data File 1 [3]. Using the well-curated profiles of the *PfamA* database [4], we constructed the architectures shown in Table 1, Data File 1 [3]. The architectures were then used to inspect the genomes of 325 Firmicutes extracted from the Kyoto Encyclopedia of Genes and Genomes (KEGG) genome database [5–7], using the *hmmscan* program from the HMMER suite [8]. We preserved those hits with an expectation value > 0.001. We discarded sequences that do not preserve the same kind of domains and with a length not longer than twice the length of the model. We used the genome neighborhood as a parameter of selection to discriminate homologous proteins not belonging to the Spo0 pathway. To this end, we inspected three genes upstream and downstream from the query gene. Frequently, neighbor genes encode proteins that were organized in a cluster of orthologous genes (COG) [9]. COGs were assigned by finding homologous proteins for each query and neighbors using a hidden Markov model (HMM) search using the *hmmsearch* program [8]. This HMM search process employs a previously constructed model set that represents each of the 4873 COGs [9, 10]. The genomes tested were annotated using Operon Mapper, which is able to classify genes into COGs [10, 11]. The list of the COGs assigned to the Spo0 proteins and their neighbors is available in Table 1, Data File 2 [12].

**Table 1:** Overview of data files/data sets

Label	Name of data file/data set	File type (file extension)	Data repository and identifier (DOI or accession number)
Data File 1 [3]	Architectures, Pfams and model organisms	MS Excel file (.xlsx)	Figshare (10.6084/m9.figshare.9701522)
Data File 2 [12]	COGs	MS Excel file (.xlsx)	Figshare (10.6084/m9.figshare.9701630)
Data File 3 [14]	Spo0 predicted pathways and curated Firmicutes	MS Excel file (.xlsx)	Figshare (10.6084/m9.figshare.9876683)

To detect orphan histidine kinases (HKo), defined as kinases not having as a neighboring response regulator [13], we performed a *hmmscan* using the profiles of the *PfamA* shown in Table 1, Data File 1 [3] and kept those hits with an expectation value > 0.001 that fulfill the architectures described in Table 1, Data File 1 [3]. As an additional condition, we discarded those sequences that did not have the same number of domains and with a length no more than twice the length of the model. As the architectures were constructed using proteins experimentally proven to participate in sporulation (Table 1, Data File 1) [3], we considered for this analysis the architecture found in *Clostridium thermocellum*, which is composed of a HisKA, an HATPase, and a response regulator domain (Table 1, Data File 1) [3].

Spo0 pathways found for each endospore former are shown in Table 1, Data File 3 [14]. These results show the architectures describing twelve HKos, three architectures representing transferases, and two architectures describing the response regulator Spo0A. The final table includes 185 Bacilli, 134 Clostridia, 4 Negativicutes, and 2 Erysipelotrichia species.

## Limitations

The data presented in this note were not published as a research article since the main findings were published while our manuscript was under review. Nonetheless, the data are still important and useful since they are a collection of well-curated Firmicutes species for which a Spo0 pathway was present or absent.

- Experimental evidence of a sporulation phenotype is not available for a vast number of strains since the conditions have not been identified.
- New protein profiles should be constructed to identify novel orphan kinases.
- A study should be performed in newly sequenced genomes to increase the collection; nonetheless, the groups presented in this note are probably enough to confirm the main conclusions found by both groups.

## Data Availability

The data described in this data note can be freely and openly accessed on Figshare under the DOIs shown in Table 1. Please see Table 1 and the reference list numbers [3, 12, 14] for details and links to the data.

## Abbreviations

HMM: hidden Markov model
COG: cluster of orthologous genes

## References

[1] Capra EJ, Laub MT (2012). Evolution of two-component signal transduction systems. Annu Rev Microbiol.

[2] Davidson P, Eutsey R, Redler B, Hiller NL, Laub MT, Durand D (2018). Flexibility and constraint: evolutionary remodeling of the sporulation initiation pathway in firmicutes. PLoS Genet.

[3] Data file 1. Proteins model architectures of the signal transduction cascade in endospore formers. 2019. 10.6084/m9.figshare.9701522.

[4] El-Gebali S, Mistry J, Bateman A, Eddy SR, Luciani A, Potter SC (2019). The Pfam protein families database in 2019. Nucleic Acids Res.

[5] Kanehisa M, Furumichi M, Tanabe M, Sato Y, Morishima K (2017). KEGG: new perspectives on genomes, pathways, diseases, and drugs. Nucleic Acids Res.

[6] Kanehisa M, Sato Y, Kawashima M, Furumichi M, Tanabe M (2016). KEGG as a reference resource for gene and protein annotation. Nucleic Acids Res.

[7] KEGG Organisms: complete genomes. https://www.genome.jp/kegg/catalog/org_list.html.

[8] Finn RD, Clements J, Arndt W, Miller BL, Wheeler TJ, Schreiber F (2015). HMMER web server: 2015 update. Nucleic Acids Res.

[9] Tatusov RL, Fedorova ND, Jackson JD, Jacobs AR, Kiryutin B, Koonin EV (2003). The COG database: an updated version includes eukaryotes. BMC Bioinform.

[10] Taboada B, Verde C, Merino E (2010). High accuracy operon prediction method based on STRING database scores. Nucleic Acids Res.

[11] Taboada B, Estrada K, Ciria R, Merino E (2018). Operon-mapper: a web server for precise operon identification in bacterial and archaeal genomes. Bioinformatics.

[12] Data file 2. COG defining the Spo0 pathway. 2019. 10.6084/m9.figshare.9701630.

[13] Dürre P (2011). Ancestral sporulation initiation. Mol Microbiol.

[14] Data file 3. Spo0 pathwaySpo0 predicted pathways and curated Firmicutes. 2019. 10.6084/m9.figshare.9876683.

